# Trends in racial and ethnic disparities in the health-related quality of life of older adults with breast cancer: a SEER-MHOS national database study

**DOI:** 10.1186/s12955-025-02359-x

**Published:** 2025-03-26

**Authors:** Nicki Karimi-Mostowfi, Fang-I Chu, Trudy C. Wu, Matthew J. Farrell, Wisdom Akingbemi, Ann C. Raldow

**Affiliations:** 1 Lewis Katz School of Medicine, Temple University, Philadelphia, PA USA; 2 UCLA Department of Radiation Oncology, David Geffen School of Medicine at UCLA, Westwood Radiation Oncology, 200 Medical Plaza, Suite B265, Los Angeles, CA 90095 USA; 3 Drexel University College of Medicine, Philadelphia, PA USA

**Keywords:** Breast cancer, HRQOL, Racial disparities, Ethnic disparities, Quality of life

## Abstract

**Purpose:**

To examine racial and ethnic disparities in Health-Related Quality of Life (HRQOL) in older adults with breast cancer, both pre- and post-diagnosis.

**Methods:**

Using the SEER-MHOS database, we included patients *≥* 65 years old with breast cancer who completed the Health Outcomes Survey within 24 months pre- and post-diagnosis, and who were non-Hispanic White, non-Hispanic Asian or Pacific Islander, non-Hispanic Black or African American, or Hispanic. HRQOL data was measured via the Physical and Mental Component Summary (PCS, MCS). Univariable and multivariable linear regression models were fitted to assess for potential disparities between races and ethnicities.

**Results:**

On univariable regression models, a numerical drop in mean scores of PCS and MCS was found among all racial/ethnic groups between pre- and post-diagnosis. Among patients in the pre-diagnosis cohort who would be diagnosed with stage IV breast cancer, race was found to be a predictor of PCS with overall significance (*p* = 0.04). On the local test, compared with Black individuals, White individuals had higher pre-diagnosis PCS scores (+ 13.32, *p* = 0.03). Race/ethnicity was not found to be a predictor in PCS or MCS scores otherwise.

**Conclusion:**

Among older patients diagnosed with stage IV breast cancer, White individuals had better physical HRQOL than Black patients’ pre-diagnosis. The decrease in the numerical HRQOL scores of the physical domain in all groups post-diagnosis highlights the potential negative physical impact of breast cancer has on patients, demonstrating the need for determining the proper resources and support to improve physical HRQOL following diagnosis.

## Introduction

In the United States, breast cancer accounted for almost one-third of all cancers diagnosed in women in 2024 and was the second leading cause of cancer death in women [1]. There are striking mortality differences between different racial and ethnic groups diagnosed with breast cancer —for example, while Black women have a 4% lower breast cancer incidence than White women, they have a 41% higher mortality rate [1]. The incidence and death rates of breast cancer are lower among women of other racial and ethnic groups, including women of Hispanic, Latino, and Spanish origin; American Indian and Alaskan Native origin; and Native Hawaiian or other Pacific Islander origin than among Black and White women [2]. The reason for this is likely multifactorial—there are many potential socioeconomic and genetic factors contributing to this disparity. For example, Black women have more barriers to healthcare access, are more likely to obtain inadequate treatment, and are more likely to have comorbidities, as compared to other groups [2]. Black women are less likely to receive a referral for mammography and less likely to have a timely follow-up after an abnormal screen [1]. Black women are also more likely to be diagnosed with more aggressive pathology including triple-negative breast cancer [3].

Physical and psychological distress is common among patients with breast cancer [4]. Nearly 50% of women with early-stage breast cancer have symptoms of depression, anxiety, or both within the first year after diagnosis [5]. Black women, particularly those with the triple-negative phenotype, experience even poorer psychological adjustment and physical health [6]. Although not specific to breast cancer, depression has a substantial impact on Health-Related Quality of Life (HRQOL) and is associated with increased healthcare costs and utilization, as well as decreased adherence to cancer treatment [7]. Given that ethnically diverse women show a greater burden of psychological consequences, it is important to determine whether there are differences in the HRQOL between different subpopulations of patients, and the extent to which they exist. While several studies have examined HRQOL differences between breast cancer patients of different ethnicities, the data are primarily focused on non-Hispanic Black and non-Hispanic White women, and inconsistences in conclusions have been drawn. Consequently, racial and ethnic disparities in the HRQOL of patients with breast cancer remain poorly understood.

Breast cancer, like most cancers, is a disease of aging, with elderly patients making up a significant portion of those affected. Despite this, there are still very few standardized guidelines for how best to treat and screen this population [8]. Furthermore, clinical trials are typically conducted on patients aged 18–64 years, with older patients being poorly represented, emphasizing the critical need to focus on the elderly population. Therefore, the objectives of this study were to examine differences in HRQOL by race and ethnicity among older adults with breast cancer, both pre-and post- diagnosis. We examined HRQOL via the Mental Component Summary (MCS) and Physical Component Summary (PCS).

## Materials and methods

### Data source

This study used data from the Surveillance, Epidemiology, and End Results (SEER)–Medicare Health Outcomes Survey (MHOS), a national tumor registry and linked data resource covering the United States population. SEER, a registry created in 1973, has statistics on 29 cancer subtypes and individual cancer patients, including incidence, mortality, survival, and prevalence. SEER-MHOS combines the SEER data with longitudinal health outcomes survey data and gathers information including but not limited to HRQOL. With SEER-MHOS, the race and ethnicity variable, as a demographic feature, was based on self-reported information from the MHOS survey [9]. If this information was unavailable, race and ethnicity data were obtained from the SEER file or the Centers for Medicare and Medicaid services database [9]. We identified patients aged 65 years and older with breast cancer who were diagnosed between 1998 and 2019 and who completed the health outcomes survey 24 or fewer months before diagnosis or after diagnosis. Individuals who were younger than 65 years at the time of the survey and those who completed the health outcomes survey more than 24 months pre- and/or post-diagnosis were excluded from the study, as were patients with missing information. Breast cancer patients with a prior cancer diagnosis were also excluded.

The PCS and MCS scores of the Medical Outcomes Study Short Form-36 and the Veterans RAND 12-Item Health Survey instruments were used to measure HRQOL. The 12-Item Health Survey included tests of Bodily Pain, General Health, Mental Health, Physical Functioning, Role-Emotional, Role-Physical, Social Functioning, and Vitality. Using both PCS and MCS has been shown to be a more reliable measure of health outcomes than either measure alone. The scores range from 0 to 100, with higher scores representing better self-reported health.

### Statistical analysis

Patients were grouped into the following race/ethnicity categories: non-Hispanic White (hereafter, White), non-Hispanic Black or African American (hereafter, Black), non-Hispanic Asian or Pacific Islander (hereafter, Asian or Pacific Islander), and Hispanic. Non-Hispanic American Indian or Alaskan Native, multiracial patients, and those patients characterized as “other” were excluded because of insufficient sample sizes. Patient characteristics were summarized using the mean (SD) and frequency (%).

The mean pre- and post-diagnosis MCS and PCS scores were calculated for each race/ethnicity group. Differences in the means and medians of MCS and PCS scores among the race/ethnicity groups were first assessed within the pre- and post-diagnosis cohort, respectively, via univariate analysis. To determine the strength of the association between race/ethnicity and HRQOL, multivariable linear regression analysis was further performed controlling for stage, age, gender, marital status, education, income, number of comorbidities, Activities of Daily Living (ADL) count, U.S. region, histology, and treatment (the treatment variable was included only in the post-diagnosis cohort). Staging was characterized in both pre- and post-diagnosis cohorts; in the pre-diagnosis cohort, patients were categorized depending on what stage they would go on to be diagnosed with.

The literature has shown that even modest differences in the PCS and MCS scores may reflect clinically meaningful differences in physical functioning and/or mental well-being among patients [10]. While absolute thresholds have been used with caution, a 3–5 point difference in scale scores have been shown to demonstrate a change [10]. These small variations may manifest in patients as changes in pain levels, mobility, cognitive function, or other aspects of health.

For multivariable linear regression analysis, the likelihood ratio test, a global test, was performed to assess the overall significance of race and treatment as categorical variables. When overall significance is present, pairwise comparisons, such as local tests, between each race types are assessed via post-hoc analysis. Sensitivity analysis was carried out for the cohort of subjects who completed both pre- and post-diagnosis surveys to assess the consistency of the results. All analyses were carried out in R [11].

## Results

### Demographics

There were 1,008 pre-diagnosis and 991 post-diagnosis patients included in this study, each unique within their respective cohort, and 305 patients having completed both pre- and post-diagnosis survey. 56.6% of patients in the pre-diagnosis cohort went on to be diagnosed with stage 1 breast cancer, 29.8% with stage II, 9.3% with stage III, and 4.3% with stage IV. Post-diagnosis, 58.3% of patients had stage I breast cancer, 28.6% had stage II, 9.3% had stage III, and 3.8% had stage IV. Post-diagnosis, 35.6% of patients received both surgery and radiation, 13.0% received surgery, chemotherapy, and radiation, and 51.4% either received other treatments or treatments that could not be determined.

Demographic comparisons, pre- and post-diagnosis, are shown in Table 1. The cohorts did not differ significantly with respect to the listed variables.

**Table 1:** Demographics comparisons of patients pre- and post-diagnosis

		Pre-diagnosis (*N* = 1008)		Post-diagnosis (*N* = 991)		*p*-value
		N	Percent	N	Percent	
Age at survey	65 *≤* age < 70	296	29.37%	283	28.56%	0.98
	70 *≤* age < 75	295	29.27%	292	29.47%	
	75 *≤* age < 80	199	19.74%	196	19.78%	
	*≥* 80	218	21.63%	220	22.20%	
Gender	Female	988	98.02%	967	97.58%	0.61
	Male	20	1.98%	24	2.42%	
Race	Hispanic	84	8.33%	76	7.67%	0.86
	Non-Hispanic - Asian or Pacific Islander	80	7.94%	82	8.27%	
	Non-Hispanic - Black or African American	109	10.81%	99	9.99%	
	Non-Hispanic - White	735	72.92%	734	74.07%	
Region	Midwest	113	11.21%	115	11.60%	0.95
	Northeast	122	12.10%	123	12.41%	
	South	246	24.40%	231	23.31%	
	West	527	52.28%	522	52.67%	
Marital Status	Never married	35	3.47%	43	4.34%	0.16
	Divorced	159	15.77%	177	17.86%	
	Married	425	42.16%	433	43.69%	
	Separated	14	1.39%	18	1.82%	
	Widowed	375	37.20%	320	32.29%	
Education	8th grade or less	66	6.55%	62	6.26%	0.55
	4 year college graduate	93	9.23%	97	9.79%	
	High school graduate or GED	353	35.02%	330	33.30%	
	More than a 4 year college degree	95	9.42%	118	11.91%	
	Some college or 2 year degree	289	28.67%	270	27.25%	
	Some high school, but did not graduate	112	11.11%	114	11.50%	
Income	Less than $5,000	57	5.65%	62	6.26%	0.97
	$10,000-$19,999	246	24.40%	236	23.81%	
	$100,000 or more	40	3.97%	46	4.64%	
	$20,000-$29,999	191	18.95%	185	18.67%	
	$30,000-$39,999	126	12.50%	125	12.61%	
	$40,000-$49,999	102	10.12%	86	8.68%	
	$5,000-$9,999	92	9.13%	93	9.38%	
	$50,000-$79,999	115	11.41%	116	11.71%	
	$80,000-$99,999	39	3.87%	42	4.24%	
Stage	1	571	56.65%	578	58.32%	0.87
	2	300	29.76%	283	28.56%	
	3	94	9.33%	92	9.28%	
	4	43	4.27%	38	3.83%	
Number of Comorbidities	0	177	17.56%	178	17.96%	0.26
	1	369	36.61%	322	32.49%	
	2	263	26.09%	275	27.75%	
	*≥* 3	199	19.74%	216	21.80%	
Number of ADL Limitations	0	642	63.69%	589	59.43%	0.25
	1	143	14.19%	150	15.14%	
	2	98	9.72%	111	11.20%	
	*≥* 3	125	12.40%	141	14.23%	
Treatment	Both Surgery and Radiation			353	35.62%	
	Received Surgery, Chemo, and Radiation			129	13.02%	
	Other or Unable to Determine			509	51.36%	

*Note 305 subjects completed both pre- and post-diagnosis surveys. The pooled cohort of pre- and post-diagnosis therefore contains 1694 unique subjects

The mean MCS and PCS scores are shown in Table 2. On univariate analyses, for both the pre- and post-diagnosis cohorts, significant differences in MCS and PCS means were identified among all race groups.

**Table 2:** Univariate analysis of mean PCS and MCS scores, Pre- and Post-Diagnosis

	Race	PCS (Mean)	PCS (SD)	MCS (Mean)	MCS (SD)
**Pre-diagnosis**	Hispanic	37.54	11.77	48.73	13.12
	Non-Hispanic Asian or Pacific Islander	40.04	10.87	52.93	10.28
	Non-Hispanic Black or African American	36.99	11.13	50.01	11.88
	Non-Hispanic White	40.32	12.00	53.06	10.24
		*P* < 0.001		*P* < 0.001	
**Post-diagnosis**	Hispanic	36.75	11.89	46.76	12.63
	Non-Hispanic Asian or Pacific Islander	39.16	10.92	51.10	10.77
	Non-Hispanic Black or African American	34.47	11.38	49.81	12.07
	Non-Hispanic White	38.50	12.06	52.26	10.62
		*P* < 0.001		*P* < 0.001	
**Difference pre-and post-diagnosis (Percent % decrease)**	Hispanic	0.79 (2.10%)		1.97 (4.04%)	
	Non-Hispanic Asian or Pacific Islander	0.88 (2.20%)		1.83 (3.46%)	
	Non-Hispanic Black or African American	2.52 (6.81%)		0.2 (0.40%)	
	Non-Hispanic White	1.82 (4.51%)		0.8 (1.51%)	

Pre-diagnosis, White individuals, with a score of 53.06 had the highest mean MCS score, followed by individuals who identified as Asian or Pacific Islander (52.93), Black (50.01), and Hispanic (48.73) (*p* < 0.001). Post diagnosis MCS followed a similar trend (52.26 vs. 51.10 vs. 49.81 vs. 46.76, respectively; *p* < 0.001). The mean PCS trends differed, with White and Asian or Pacific Islander individuals still having the highest pre-diagnosis scores (40.32 and 40.04, respectively), but with Hispanic individuals having higher scores than Black individuals (37.54 and 36.99, respectively) (*p* < 0.001). In the post-diagnosis cohort, individuals who identified as Asian or Pacific Islander had the highest PCS scores (39.16), followed by White (38.50), Hispanic (36.75), and Black (34.47) individuals.

The differences in scores from pre- to post-diagnosis are also shown in Table 2. In terms of PCS scores, the largest decrease was seen in Black patients (6.81% decrease), and in terms of MCS scores, the largest decrease was observed in Hispanic patients (4.04% decrease). The smallest difference in scores pre- and post-diagnosis was observed in Black patients in MCS (0.4% decrease).

### Pre-diagnosis multivariable linear regression analyses

On multivariable linear regression analysis, regarding PCS scores in the pre-diagnosis cohort (Table 3), increased age (*p* < 0.01), number of comorbidities (*p* < 0.001), and ADL limitations (*p* < 0.001) had lower scores. For the MCS scores (Table 3), a higher ADL limitation count (*p* < 0.001) and the male gender (*p* < 0.01) were associated with lower MCS scores. Income levels of $50,000 to $79,000 (*p* = 0.03) and $100,000 or more (*p* = 0.04) were associated with higher MCS scores than patients income levels of less than $5,000, and older patients also had significantly higher MCS scores (*p* < 0.01).

**Table 3:** PCS and MCS multivariable analysis in the Pre-Diagnosis cohort

Characteristics	PCS	PCS *p*-value	MCS	MCS *p*-value
Race				
Reference: Hispanic				
Non-Hispanic Asian or Pacific Islander	-1.12	0.46	-0.60	0.71
Non-Hispanic Black or African American	-0.25	0.86	2.64	0.09
Non-Hispanic White	-0.92	0.43	1.27	0.31
Stage				
Reference: T1/T2				
T3/T4	-1.37	0.12	1.30	0.16
Age	-0.14	< 0.01	0.15	< 0.01
Gender				
Reference: Female				
Male	0.26	0.90	-6.75	< 0.01
Marital Status				
Reference: Never married				
Divorced	-1.18	0.51	-1.05	0.58
Married	-1.82	0.29	-2.22	0.22
Separated	-0.80	0.79	-4.29	0.18
Widowed	-0.25	0.88	-2.03	0.26
Educational Level				
Reference: 8th grade or less				
4-year college graduate	2.17	0.18	3.20	0.06
High school graduate or GED	0.64	0.63	2.35	0.10
More than a 4-year college degree	1.57	0.34	1.31	0.46
Some college or 2-year degree	1.39	0.32	2.57	0.08
Some high school, but did not graduate	0.16	0.92	1.77	0.26
Income				
Reference: <$5,000				
$5,000-$9,999	-2.71	0.09	-2.16	0.21
$10,000-$19,999	-1.16	0.41	0.06	0.97
$20,000-$29,999	0.00	1.00	0.85	0.59
$30,000-$39,999	-0.19	0.90	1.46	0.38
$40,000-$49,999	0.67	0.68	0.24	0.89
$50,000-$79,999	2.54	0.12	3.86	0.03
$80,000-$99,999	-0.49	0.81	4.04	0.07
$100,000 or more	3.20	0.13	4.66	0.04
Comorbidities	-2.15	< 0.001	-0.31	0.24
ADL Count	-3.93	< 0.001	-2.43	< 0.001
Region				
Reference: Midwest				
Northeast	-0.32	0.80	1.76	0.19
South	-2.14	0.05	1.13	0.33
West	-1.01	0.31	1.82	0.09
Histology				
Reference: Non-Adenomas and Non-Adenocarcinomas				
Adenomas and Adenocarcinomas	0.28	0.86	0.87	0.60

**Table 4:** PCS and MCS multivariable analysis in the Post-Diagnosis cohort

Characteristics	PCS	PCS *p*-value	MCS	MCS *p*-value
Race				
Reference: Hispanic				
Non-Hispanic Asian or Pacific Islander	2.21	0.15	0.28	0.87
Non-Hispanic Black or African American	0.15	0.92	3.83	0.02
Non-Hispanic White	0.24	0.85	1.80	0.19
Treatment				
Reference: Surgery and Radiation				
Other or Unable to Determine	-0.27	0.70	0.30	0.69
Surgery, Chemo and Radiation	-1.01	0.33	0.50	0.66
Stage				
Reference: T1/T2				
T3/T4	-0.82	0.38	2.07	0.04
Age	-0.07	0.18	0.18	< 0.01
Gender				
Reference: Female				
Male	2.05	0.31	-0.89	0.69
Marital Status				
Reference: Never married				
Divorced	-0.84	0.61	1.02	0.57
Married	-1.26	0.42	-0.24	0.89
Separated	-0.51	0.85	2.97	0.32
Widowed	-0.65	0.68	0.70	0.69
Educational Level				
Reference: 8th grade or less				
4-year college graduate	-0.58	0.73	5.30	< 0.01
High school graduate or GED	-2.04	0.15	4.37	< 0.01
More than a 4-year college degree	-1.63	0.33	3.21	0.08
Some college or 2-year degree	-2.07	0.16	2.78	0.08
Some high school, but did not graduate	-2.60	0.09	1.39	0.41
Income				
Reference: <$5,000				
$5,000-$9,999	2.17	0.17	3.00	0.08
$10,000-$19,999	2.06	0.14	3.06	< 0.05
$20,000-$29,999	2.60	0.08	4.36	< 0.01
$30,000-$39,999	1.85	0.24	5.09	< 0.01
$40,000-$49,999	2.70	0.11	4.58	0.01
$50,000-$79,999	2.47	0.14	7.84	< 0.001
$80,000-$99,999	3.53	0.09	6.71	< 0.01
$100,000 or more	6.04	< 0.01	7.04	< 0.01
Comorbidities	-1.96	< 0.001	-0.19	0.46
ADL Count	-3.74	< 0.001	-2.61	< 0.001
Region				
Reference: Midwest				
Northeast	1.37	0.28	-0.62	0.65
South	0.85	0.44	-0.81	0.50
West	0.51	0.62	0.50	0.66
Histology				
Reference: Non-Adenomas and Non-Adenocarcinomas				
Adenomas and Adenocarcinomas	-1.30	0.42	0.82	0.64

Among patients in the pre-diagnosis cohort who would be diagnosed with stage IV breast cancer, multivariable regression revealed that race was a significant predictor of PCS (*p* = 0.04). Compared with Black patients, White patients had higher pre-diagnosis PCS scores (+ 13.32, *p* = 0.03). No further statistically significant differences with respect to race/ethnicity were found. The subcohorts of Stage I, II, and III breast cancer patients had no significant findings regarding race or ethnicity and thus are not reported.

Considering overall significance, race and ethnicity were not associated with PCS or MCS scores for the entire cohort or any other stage as subcohort.

### Post-diagnosis multivariable linear regression analyses

Within the entire post-diagnosis cohort regarding PCS scores (Table 4), neither race nor treatment was found to be a predictor with overall significance. The PCS scores decreased with increasing ADL limitations (*p* < 0.001) and number of comorbidities (*p* < 0.001). Furthermore, compared with patients with an income level of less than $5,000, patients with an income level of more than $100,000 had higher PCS scores (*p* < 0.01).

Within the entire post-diagnosis cohort, the MCS scores displayed a different trend. Race was not found to be a predictor of MCS with overall significance (*p* = 0.10), with local test indicating that Black patients had higher MCS scores than Hispanic patients did (*p* = 0.02). Older age (*p* < 0.01) and cancer stage 3 to 4, compared to stage 1 to 2 (*p* = 0.04) had higher MCS scores. Compared with those with an educational level of 8th grade or less, 4-year college graduates (*p* < 0.01) and high school graduates or GED holders (*p* < 0.01) had higher MCS scores. Furthermore, compared with income levels of less than $5,000, all the following had higher MCS scores: $10,000 to $19,999 (*p* < 0.05), $20,000 to $29,999 (*p* < 0.01), $30,000 to $39,999 (*p* < 0.01), $40,000 to $49,999 (*p* = 0.01), $50,000 to $79,999 (*p* < 0.001), $80,000 to $99,999 and $100,000 and more (*p* < 0.01, *p* < 0.01). On the other hand, those with greater ADL limitations (*p* < 0.001) had lower MCS scores.

The MCS scores of the Stage I subcohort were similar to those of the entire post-diagnosis cohort. Local tests indicated that, compared to Hispanic patients, Black and White patients had higher MCS scores (*p* = 0.02, *p* = 0.01). However, race was not found to be a predictor of MCS with overall significance (*p* = 0.06). The subcohorts of Stages II, III, and IV had no significant findings regarding race or ethnicity and thus are not reported.

With sensitivity analysis among subjects who had completed both surveys, univariate analysis revealed that the identified difference in race become unfound, which may be due to the relatively smaller sample size (*n* = 305) in this sub-cohort, compared to pre- and post- diagnosis cohorts. On multivariable analysis, overall, the findings for this subcohort are similar as with pre- and post- diagnosis cohorts, respectively. These findings indicate that the results are consistent.

## Discussion

In this study, Health Related Quality of Life (HRQOL) was examined using Mental (MCS) and Physical (PCS) Component Summary domains. Among patients aged 65 years or older who received a diagnosis of stage IV breast cancer, White patients had better physical HRQOL compared to Black patients’ pre-diagnosis (*p* = 0.04). Within the entire pre- and post-diagnosis cohort, significant differences in MCS and PCS scores were found among the racial groups; the trend of a decrease in scores from before to after diagnosis was consistently observed. However, after adjusting for potential confounding variables in multivariable linear regression, race and ethnicity were not found to be predictors of HRQOL scores with overall significance. This lack of overall significance also holds true in the two instances where local tests indicated that race was correlated with a difference in MCS scores (within the entire post-diagnosis cohort and in the stage I subcohort).

In pre-diagnosis PCS and post-diagnosis MCS and PCS, a greater number of patient comorbidities and ADL limitations had lower scores. The only domain in which this was not observed was the pre-diagnosis MCS scores. Income, age, education level, region, cancer stage, and gender had varying effects on scores, with some not showing any effect at all. Higher income levels were inclined to have higher MCS and PCS scores, although this was not an absolute.

Inconsistent findings regarding HRQOL differences between breast cancer patients of different ethnicities have been noted in prior studies and have focused mainly on White and Black patients. For example, in a study by Ye et al., it was found that when adjusting for demographic features and comorbid conditions, the MCS score, but not the PCS score, was lower for Black patients than White patients [12]. In another study by Ashing-Giwa et al., no differences in quality-of-life outcomes were attributed to ethnicity [13]. Two studies by Paskett et al. and Bowen et al. reported that Black women with breast cancer had decreases in physical HRQOL and lower physical functioning scores, respectively, compared with their White counterparts [14, 15]. While the results of our study contradict those of previous studies, there are key points in these studies that differ from ours. All three studies only included or solely assessed patients younger than 65 years, only evaluated patients who were White or Black, and only examined scores post-diagnosis.

Decreases in MCS and PCS scores are associated with increased all-cause mortality in patients and HRQOL assessments at the time of diagnosis can help to predict survival in patients [16, 17]. The decrease in HRQOL scores in all races and ethnicities from pre- to post-diagnosis suggests that regardless of race and ethnicity, physical distress, psychological distress, and the mental toll of breast cancer have a strong effect on individuals, necessitating better resources, treatment, and support. Patients can benefit from early intervention to increase their chances of survival and improve their quality of life after treatment. While there were differences in univariable linear regression analyses such that Hispanic patients had the largest decrease in MCS scores and Black patients had the largest decrease in PCS scores but the smallest decrease in MCS scores, these differences do not seem to be attributable to race due to the lack of differences in multivariable linear regression. Any resource, treatment, or support could have a positive impact on all patients within similar situations (income, education, etc.), regardless of race and ethnicity. Additionally, while the reasoning for the MCS scores in Black patients is not definitive, the smaller decrease could be in part due to different cultural practices among Black patients, namely regarding their increased spirituality and sense of community [18]. Providing resources to women of other races to form support groups similar to Black patients could prove to be advantageous, but further research is needed. Furthermore, medical care should focus on reducing patient comorbidities and increasing feasible ADLs across all races and ethnicities, as a lower number of comorbidities and greater ability to perform ADLs are significantly correlated with higher MCS and PCS scores in most domains.

A strength of this study was the use of SEER linked data; this database gives us access to a large and high-quality dataset from across the United States and allows us to compare individuals pre- and post-diagnosis. We were also able to use local tests to determine significant differences between grouped variables as well as global tests to determine the overall effect of race/ethnicity inclusion on HRQOL changes. We were also able to analyze an understudied population —individuals aged 65 + years old—although a limitation would then be that the results are not necessarily generalizable to the general population. While a study advantage was that the pre- and post-diagnosis cohort had similar sample sizes, further limitations to this study include differences in sizes among racial and ethnic groups. White patients made up the bulk of the sample size, with approximately 740 patients pre- and post-diagnosis, compared with approximately 80 for Hispanic and API patients, and 100 for Black patients. This shows a stronger reporting power for the White patient cohort. Many subjects were excluded from the study, even if they were missing only one variable, which significantly reduced the sample population size. Furthermore, the conflicting results on race and treatment obtained from the likelihood ratio test as the global test and local test may be due to the present but unaddressed multiple testing issue of the local test, which requires further validation and investigation. Additionally, not all data are captured in the SEER-MHOS database, which is limited to Medicare enrollees only. Therefore, the results may not be generalizable to those who have different insurance policies. Finally, the difference in race observed in the univariate analysis suggests the presence of confounding factors. In the multivariable analysis, the association between survey scores and race was no longer significant after adjusting for potential confounders. These confounding factors may include comorbidities, ADL counts, income, and other variables. Future studies should aim to account for differences in general living conditions and overall quality of life to minimize the impact of confounding.

Using the national SEER-MHOS, we determined that, when adjusting for potential confounders, mental and physical HQROL scores decrease for all racial and ethnic groups. Additionally, disparities in overall scores exist only among the different racial/ethnic groups in Stage IV breast cancer patients at pre-diagnosis. Limitations in ADLs and patient comorbidities were the only variables that almost always had a decrease in scores across stages and racial/ethnic groups. As a result, further studies should aim to evaluate how different races and ethnicities cope with their breast cancer and assess the effects of targeting the comorbidities and ADLs of patients on overall HRQOL and specific HRQOL domains. To our knowledge, this is the first study to present such HRQOL considerations while evaluating breast cancer patients.

## Data Availability

The datasets generated during and/or analyzed during the current study are not publicly available but are available to outside investigators for research purposes. Investigators are required to obtain approval in order to obtain the data.

## Abbreviations

HRQOL: Health-Related Quality of Life
PCS: Physical Component Summary
MCS: Mental Component Summary
SEER-MHOS: Surveillance, Epidemiology, and End Results (SEER)–Medicare Health Outcomes Survey (MHOS)
ADL: Activities of Daily Living
